# The role of cognitive function in age‐related changes in spatiotemporal gait: findings from an observational study

**DOI:** 10.1002/alz70856_102900

**Published:** 2025-12-26

**Authors:** Ran An

**Affiliations:** ^1^ Peking University, Beijing, China

## Abstract

**Background:**

Gait performance decline is commonly observed in older adults with cognitive impairment. However, the extent to which cognitive function influences gait, beyond the effects of aging, remains unclear. To investigate the trajectory of spatiotemporal gait across the dementia spectrum with aging and identify the mediating role of cognitive function in the relationship between aging and gait performance.

**Method:**

This observational study analyzed data from an ongoing cohort study conducted in China starting in 2022. Of 447 eligible individuals from community settings, 289 participated and were categorized into three groups based on clinical diagnosis: 64 cognitively normal (CN), 168 with subjective cognitive decline (SCD), and 77 with cognitive impairment (CI). Eligible participants underwent neuropsychological and gait assessments under both single‐task and dual‐task conditions. Gait features were assessed using computer vision, including spatial parameters, temporal parameters, and spatio‐temporal parameters. Polynomial regression models to generate age‐related trajectories of gait features. Structural equation models to evaluate the mediation effects on the relationship between biological age and gait features.

**Result:**

Among the 289 participants (mean [SD] age, 70.65 [7.76] years; 180 [62.98%] women), significant differences in stride speed were observed across the three groups under both single‐task (Kruskal‐Wallis chi‐squared statistic = 13.204, *p* < .001) and dual‐task conditions (16.357, *p* < .001). Gait trajectories exhibited both linear and nonlinear associations with biological age. Spatial and spatio‐temporal gait components progressively declined with age in all groups, while temporal parameters remained stable in the CN group but increased with age in the SCD and CI groups. Global cognitive function partially mediated the relationship between age and spatial components (stride length, standardized β=0.01, *p* < .001) and spatio‐temporal gait features (gait speed, 0.01, *p* < .001), and fully mediated the relationship between age and temporal gait features (stride time, ‐0.01, *p* < .001).

**Conclusion:**

Spatial and spatio‐temporal gait appear to be primarily influenced by normal aging, whereas temporal components are more affected by cognitive function. Gait speed serves as a non‐cognitive biomarker of cognitive decline, with prognostic potential primarily linked to temporal components.

